# PDI regulates seizure activity via NMDA receptor redox in rats

**DOI:** 10.1038/srep42491

**Published:** 2017-02-15

**Authors:** Ji Yang Kim, Ah-Rhem Ko, Hye-Won Hyun, Su-Ji Min, Ji-Eun Kim

**Affiliations:** 1 Department of Anatomy and Neurobiology, Institute of Epilepsy Research, College of Medicine, Hallym University, Chuncheon 24252, South Korea

## Abstract

Redox modulation of cysteine residues is one of the post-translational modifications of N-methyl-D-aspartate receptor (NMDAR). Protein disulfide isomerases (PDI), an endoplasmic reticulum (ER) chaperone, plays a crucial role in catalyzing disulfide bond formation, reduction, and isomerization. In the present study, we found that PDI bound to NMDAR in the normal hippocampus, and that this binding was increased in chronic epileptic rats. *In vitro* thiol reductase assay revealed that PDI increased the amount of thiols on full-length recombinant NR1 protein. PDI siRNA, 5–5′-dithio-bis(2-nitrobenzoic acid) (DTNB), bacitracin and PDI antibody reduced seizure susceptibility in response to pilocarpine. In addition, PDI knockdown effectively ameliorated spontaneous seizure activity in chronic epileptic rats. Anticonvulsive effects of PDI siRNA were correlated to the reduction of the amount of free- and nitrosothiols on NMDAR, accompanied by the inhibition of PDI activity. However, PDI knockdown did not lead to alteration in basal neurotransmission or ER stress under physiological condition. These findings provide mechanistic insight into sulfhydration of disulfide bonds on NMDAR by PDI, and suggest that PDI may represent a target of potential therapeutics for epilepsy, which avoids a possible side effect on physiological receptor functionality.

Epilepsy is a common neurological disorder characterized by a paroxysmal excitatory activity with a prevalence of 4–10 per 1000 of the population^1^. The repeated seizures of epilepsy are associated with increased mortality and the risk of psychiatric disorders and cognitive dysfunction^2^. The N-methyl-D-aspartate subtype of the glutamate receptor (NMDAR) contributes to multiple physiological and pathological processes including seizures generation and epileptogenesis^3^,^4^,^5^,^6^. The binding of endogenous modulators (e.g. glycine, Zn^2+^, Mg^2+^ and D-serine) to NMDAR regulates the channel activity^7^,^8^,^9^. Similar to receptor ligand-modulators, redox modulation of cysteine residues is one of the post-translation NMDAR modifications, which induces the conformational changes in the NMDAR function^10^,^11^. Disulfide reductants (S-S → HS- + -SH), such as dithiothreitol (DTT) or dihydrolipoic acid, increase NMDAR-opening frequency. In contrast, free thiol oxidants like 5–5′-dithio-bis(2-nitrobenzoic acid) (DTNB) or oxidized glutathione (GSSG) inhibit NMDAR-mediated currents by disulfide formation (HS + SH → S-S)^10^,^12^,^13^. Under pathophysiological conditions, endogenous oxidizing agents (e.g. GSSG, NO and Zn^2+^) inhibit NMDAR overactivation and control NMDAR-mediated Ca^2+^ influx, thus consequently ameliorate NMDAR-mediated neurotoxicity^14^,^15^. Furthermore, DTT induces spontaneous epileptiform discharges^12^, while DTNB suppresses seizure activity^16^. Therefore, the regulation of NMDAR redox sites is a kind of the potential therapeutic antiepileptic and neuroprotective strategies. However, the endogenous reducing molecules for NMDAR redox sites are less known.

Protein disulfide isomerase (PDI) is one of the initial endoplasmic reticulum (ER) chaperones^17^,^18^. PDI is also a redox-active enzyme characterized by a thioredoxin (Trx)-like Cys-Xaa-Xaa-Cys (CXXC) catalytic domain, which plays a crucial role in catalyzing disulfide bond formation, reduction, and isomerization^19^,^20^,^21^,^22^. Recently, we have reported that PDI expression in dentate granule cells was transiently increased following status epilepticus (SE, a prolonged seizure activity) induced by pilocarpine (PILO), and restored to basal level at 4 weeks after SE^23^. With respect to these profiles of PDI, we hypothesized that PDI may be one of endogenous reducing factors for the disulfide bonds of NMDAR redox sites, and the consequent potentiation of NMDAR function could increase seizure susceptibility and sustain seizure activity. In the present study, therefore, we investigated whether endogenous PDI modulates NMDAR redox state, which contributes ictogenesis *in vivo.* Here, we demonstrate that PDI bound to NR1 and NR2A subunits. PDI knockdown decreased thiols (in other word, increased disulfide bond formation) on NMDAR under normal condition. PDI knockdown also reduced seizure susceptibility in response to PILO without alterations in GABAergic or glutamatergic transmission and ER stress induction. *In vitro* thiol reductase assay using full-length recombinant NR1 protein revealed that PDI increased the amount of thiols on NR1 subunit. Furthermore, PDI neutralization decreased seizure susceptibility in response to PILO. This anticonvulsive effect of PDI knockdown was similar to that of oxidizing agent and PDI inhibitor (DTNB and bacitracin). More important, PDI knockdown reduced the frequency and duration of spontaneous seizure activity in chronic epileptic animals. Therefore, our findings suggest that PDI may involve the dynamic redox regulation of NMDAR function, which is a critical mechanism in the seizure susceptibility, and may represent a target of potential therapeutics for epilepsy.

## Results

### PDI knockdown reduces seizure susceptibility in response to PILO

**Figure 1:**
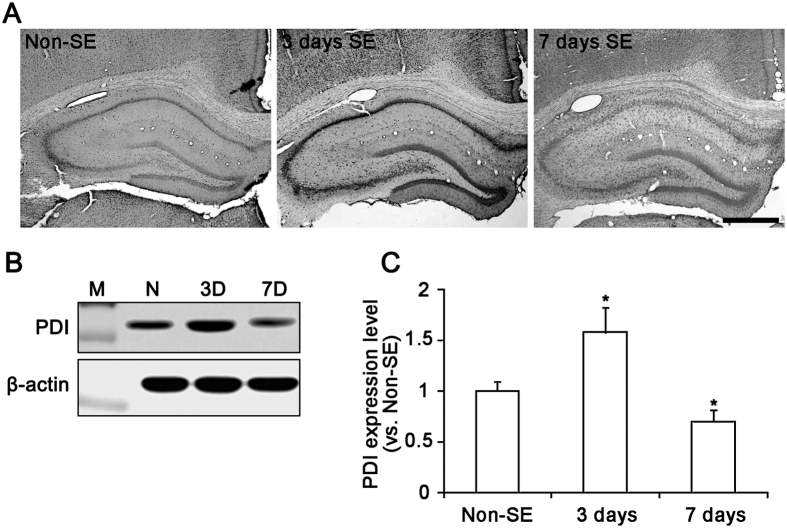
PDI expression in the hippocampus following SE. (**A**) In non-SE animals (N), PDI expression is observed in CA1–CA3 pyramidal cells as well as dentate granule cells. Three days (3D) after SE, PDI expression is increased in the same regions. However, PDI expression is decreased 7 days (7D) after SE. Bar = 300 μm. (**B**) Western blot shows the gradual up-regulation of PDI 3 days after SE. At 7 days after SE, the expression is reduced. M, molecular weight marker. (**C**) Quantification of PDI expression level based on western blot data (mean ± S.E.M.; *p < 0.05 vs. non-SE; n = 7, respectively).

Consistent with our previous study^23^, PDI expression was observed in CA1-CA3 pyramidal cells as well as dentate granule cells in non-SE animals (Fig. 1A). Three days after SE, PDI expression was transiently up-regulated in CA1-CA3 pyramidal cells and dentate granule cells. Western blot study revealed that PDI expression was increased to 2.3-fold of non-SE animals 3 days after SE (p < 0.05; Fig. 1B,C). However, PDI expression was decreased to 0.7-fold of non-SE level 1 week after SE (p < 0.05; Fig. 1B,C).

**Figure 2:**
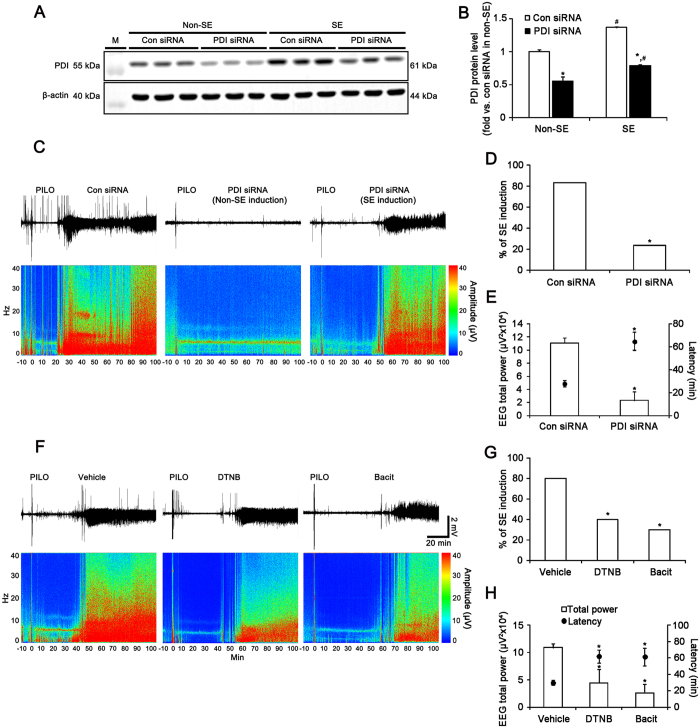
Role of PDI in seizure activity in response to PILO. (**A**) Western blot of PDI protein expression level in control siRNA- and PDI siRNA-infused animals 3 days after S.E.M., molecular weight marker. (**B**) Quantification of PDI expression level 3 days after SE (mean ± S.E.M.; *, ^#^*p* < 0.05 vs. control siRNA and non-SE animals, respectively; n = 7, respectively). PDI siRNA effectively decreases PDI protein expression level in the hippocampus. (**C–E**) Effect of PDI siRNA on seizure susceptibility in response to PILO. PDI knockdown reduces seizure susceptibility and its severity in response to PILO. (**C**) Representative EEG traces and frequency-power spectral temporal maps in response to PILO. (**D,E**) Quantification of effect of PDI siRNA on SE induction, latency and total EEG power in response to PILO (mean ± S.E.M.; *p < 0.05 vs. control siRNA; n = 30, respectively). (**F–H**) Effects of DTNB and bacitracin on seizure susceptibility in response to PILO. DTNB and bacitracin also decreases seizure susceptibility in response to PILO. (**F**) Representative EEG traces and frequency-power spectral temporal maps in response to PILO. (**G,H**) Quantification of effects of DTNB and bacitracin on SE induction, latency and total EEG power in response to PILO (mean ± S.E.M.; *p < 0.05 vs. vehicle; n = 10, respectively).

To elucidate whether the transient up-regulation of PDI overexpression plays an anti-convulsive or pro-convulsive role, we investigated the effect of PDI knockdown on seizure susceptibility in response to PILO. PDI siRNA infusion resulted in an approximate 45% reduction of PDI protein expression level in the hippocampus (p < 0.05 vs. control siRNA, Fig. 2A,B). In control siRNA (non-targeting)-infused animals, PILO induced SE in 83.3% of the total rats (n = 25 out of 30). EEG analyses revealed that typical SE, comprising episodes of high-amplitude and high-frequency discharges, occurred 28 min after PILO injection (Fig. 2C–E). Unexpectedly, PILO developed SE in 23.3% (n = 7 out of 30) of animals from PDI knockdown group (p < 0.05, vs. control siRNA; Fig. 2C–E). In PDI siRNA-infused group, SE was detected 65 min after PILO injection (p < 0.05, vs. control siRNA; Fig. 2C–E). Furthermore, PDI knockdown reduced total EEG power during SE to 0.21-fold of control siRNA infusion (p < 0.05 vs. control siRNA, Fig. 2C–E). Three days after SE, PDI expression in SE-experienced PDI knockdown animals was less up-regulated than that in control siRNA-infused animals (p < 0.05; Fig. 2A,B).

To further confirm the role of PDI in seizure activity, we investigated the effects of DTNB and bacitracin on seizure susceptibility in response to PILO. In vehicle-treated animals, 80% (n = 8 out of 10) of the total animals were developed to SE induced by PILO (Fig. 2F–H). In DTNB- and bacitracin-infused groups, 40% (n = 4 out of 10) and 30% (n = 3 out of 10) of the total animals administered PILO developed SE, respectively (p < 0.05 vs. vehicle; Fig. 2F–H). The latencies of SE on-set in vehicle-, DTNB- and bacitracin-treated animals were 30, 61 and 62 min, respectively (p < 0.05 vs. vehicle; Fig. 2F–H). In addition, total EEG powers during SE in DTNB- and bacitracin-treated animals were 0.45- and 0.26-fold of vehicle-treated animals, respectively (p < 0.05 vs. vehicle; Fig. 2F–H). Taken together, these findings indicate that PDI may play a proconvulsive role in response to PILO.

### PDI-mediated NMDAR redox regulates the seizure susceptibility in response to PILO

**Figure 3:**
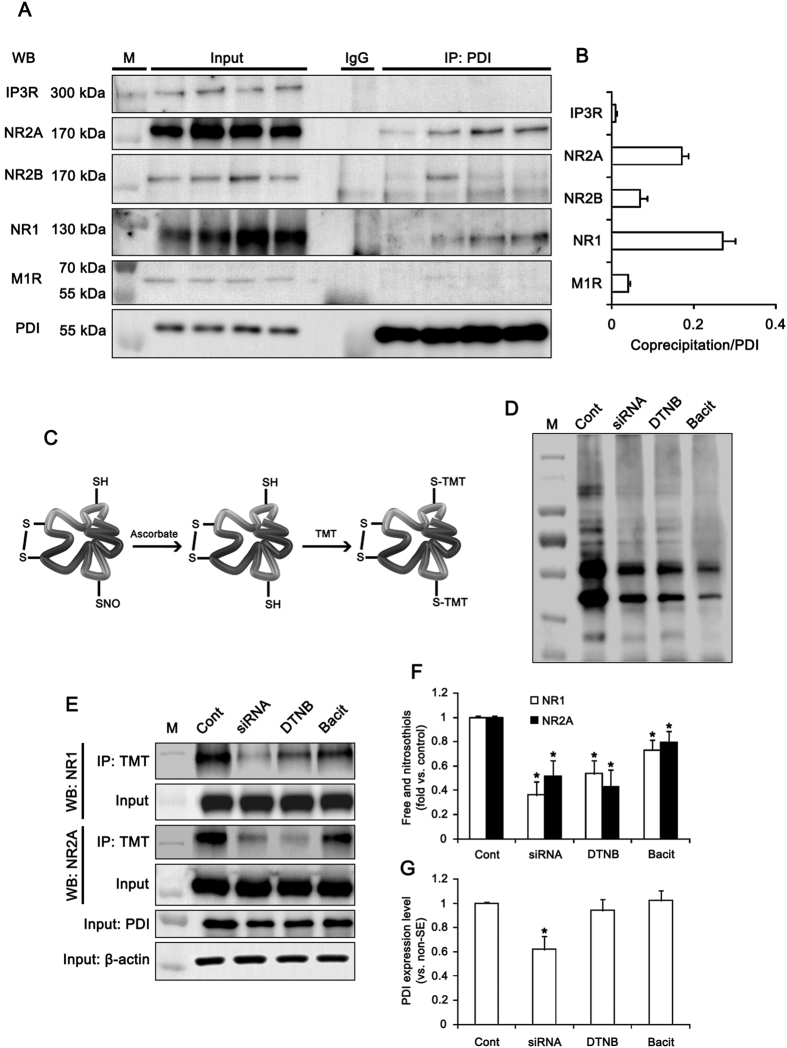
The role of PDI in redox status of NMDAR. (**A**) Co-immunoprecipitation analyses of PDI interaction with IP3R, NR2A, NR2B, NR1 and M1R in the hippocampus. M, molecular weight marker. (**B**) The quantitative analyses of co-immunoprecipitation of PDI with NR1, NR2A, NR2B, M1R and IP3. PDI binds to NR1 and NR2A more than NR2B. (**C**) Schematics of modified biotin switch technique for the measurement of the amount of free thiols (-SH) and S-nitrosothiols (-SNO). (**D**) Representative western blot for the amount of -SH + -SNO in total protein extracts. M, molecular weight marker. (**E–G**) Effects of PDI siRNA, DTNB and bacitracin on thiols on NR1 and NR2. The amount of -SH + -SNO on NR1 and NR2A subunits are significantly reduced by PDI siRNA, DTNB and bacitracin. (**E**) Western blot representing the amount of -SH + -SNO on NR1 and NR2A subunit. M, molecular weight marker. (**F,G**) Quantification of effects of PDI siRNA, DTNB and bacitracin on the amount of -SH + -SNO on NR1 and NR2A subunit and PDI expression (mean ± S.E.M.; *p < 0.05 vs. control; n = 7, respectively).

Next, we assessed whether the interaction of PDI with receptor proteins associates with the PILO-induced seizure activity. Under physiological condition, co-immunoprecipitation data revealed that PDI bound to NR1 and NR2A more than NR2B. PDI rarely bound to muscarinic acetylcholine 1 receptor (M1R) and inositol-1,4,5 trisphosphate receptor (IP3R; Fig. 3A,B). Since PDI plays a crucial role as a redox-active enzyme^19^,^20^,^21^,^22^, these findings indicate that PDI may modulate the redox sites on NMDAR, which potentiate NMDAR function^12^,^13^,^16^. To confirm this hypothesis, we measured the amount of free thiols (-SH) and S-nitrosothiols (-SNO) on NMDAR by the modified biotin-switch technique^24^ (Fig. 3C). As compared to control siRNA, PDI siRNA effectively decreased the amount of -SH + -SNO in total protein extracts (Fig. 3D). Both DTNB and bacitracin also declined it (Fig. 3D). Similar to total protein extracts, the amounts of -SH + -SNO on NR1 and NR2A subunits were significantly reduced by PDI siRNA, DTNB and bacitracin (p < 0.05 vs. vehicle or control siRNA; Fig. 3E,F), although only PDI siRNA reduced PDI expression level (p < 0.05 vs. control siRNA; Fig. 3E,G). Since -SH on cysteines of the NR1 and NR2A subunits undergoes S-nitrosylation (-SNO, nitrosothiols) or further oxidation to disulfide bonds (S-S)^11^,^24^, these decreases in the amounts of -SH + -SNO residues on NR1 and NR2A subunits represent the increase in disulfide bond formation on the redox sites of NMDAR. Therefore, our findings indicate that PDI may reduce disulfide bonds on redox status of NMDAR, which increase seizure susceptibility.

### PDI directly regulates the thiol modification on NMDAR

**Figure 4:**
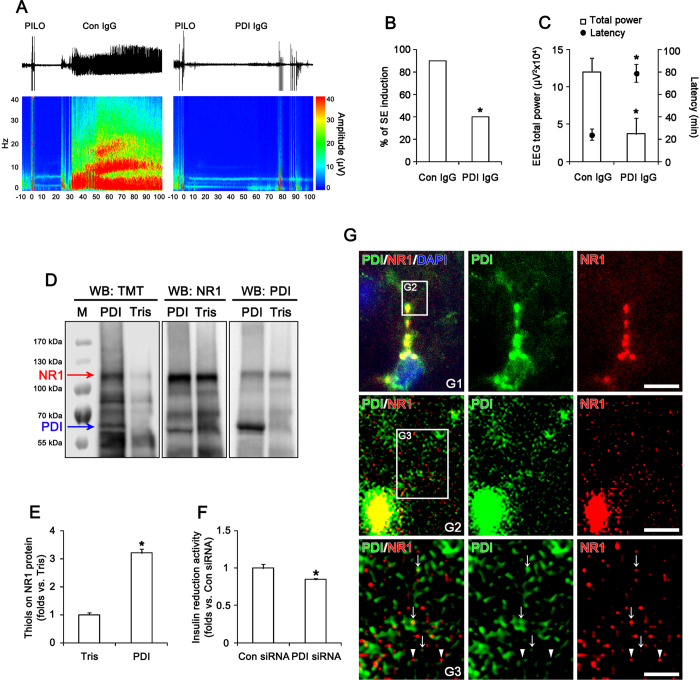
Effect of PDI neutralization on seizure activity and *in vitro* thiol reductase activity of PDI on NR1 subunit. (**A–C**) Effect of PDI neutralization on seizure susceptibility in response to PILO. PDI neutralization reduces seizure susceptibility in response to PILO. (**A**) Representative EEG traces and frequency-power spectral temporal maps in response to PILO. (**B,C**) Quantification of effect of PDI neutralization on SE induction, latency and total EEG power in response to PILO (mean ± S.E.M.; *p < 0.05 vs. control IgG; n = 10, respectively). (**D,E**) *In vitro* thiol reductase activity of PDI on recombinant NR1 protein. As compared to vehicle (Tris), PDI treatment significantly increases the amount of thiols on recombinant NR1 protein. (**D**) Western blot representing the amount of thiols on recombinant NR1 protein (the upper arrow). TMT antibody also detects the thiols on PDI (the lower arrow). M, molecular weight marker. (**E**) Quantification of effects of PDI on the amount of thiols on recombinant NR1 protein (mean ± S.E.M.; *p < 0.05 vs. vehicle; n = 7, respectively). (**F**) Effect of PDI siRNA on PDI activity *in vivo* (mean ± S.E.M.; *p < 0.05 vs. control siRNA; n = 7, respectively). PDI siRNA inhibits the insulin reduction activity, as compared to control siRNA. (**G**) Representative double immunofluorescent photo for colocazation of PDI with NR1 in the normal rat hippocampus. PDI is colocalized with NR1 clusters in perikarya and the primary dendrite (panel 1). In the distal dendrites (panel 2–3), PDI immunoreactive structures are observed as continuous tubular (ER-like) or punctuate (vesicle-like) shapes. Some PDI positive structures contain NR1 immunoreactivity (arrows) and attach to NR1 positive puncta (arrow heads). Panels 2 and 3 are high magnification images for rectangles in panels 1–2. Bar = 10 (panel 1), 5 (panel 2) and 2.5 (panel 2) μm.

Since DTNB does not easily permeate membranes^13^, DTNB-mediated NMDAR redox implicates that cell-surface or secreted PDI may reduce disulfide bonds on cysteine residues in NMDAR. To confirm whether cell-surface or secreted PDI is involved in NMDAR-mediated neuronal excitability, we investigated the presence of PDI in cerebrospinal fluid (CSF) and the effect of PDI neutralization by a specific PDI antibody on seizure susceptibility in response to PILO. Although we failed to detect PDI in CSF (data not shown), we found that PDI neutralization increased latency of seizure on-set and reduced total EEG power during SE. In control IgG-infused animals, PILO induced SE in 90% of the total rats (n = 9 out of 10; Fig. 4A,B). EEG analyses revealed the seizure on-set 24 min after PILO injection (Fig. 4A,C). PDI neutralization reduced the incidence of SE to 40% (n = 4 out of 10; p < 0.05 vs. control IgG; Fig. 4B). In PDI-treated group, SE was detected 79 min after PILO injection (p < 0.05 vs. control IgG; Fig. 4A,C). PDI neutralization decreased total EEG power during SE to 0.31-fold of control IgG (p < 0.05 vs. control IgG; Fig. 4C). These findings indicate that cell-surface PDI may be also involved in NMDAR-mediated ictogenesis.

To confirm the direct thiol reductase activity of PDI on NMDAR, we performed *in vitro* thiol reductase assays using full-length recombinant NR1 protein. PDI treatment significantly increased the amount of thiols on recombinant NR1 protein to 3.2-fold, as compared to vehicle (p < 0.05 vs. vehicle; Fig. 4D,E). *In vivo* study revealed that PDI siRNA inhibited the insulin reduction activity to 0.85-fold, as compared to control siRNA (p < 0.05 vs. control siRNA; Fig. 4F). In control (non-SE) animals, PDI immunoreactivity was found in the cytoplasm, particularly in the perinuclear region, and extended throughout the dendrites of hippocampal neurons (Fig. 4G). PDI immunoreactivity was strongly observed in perikarya and the primary dendrite (the main dendritic shaft). In these regions, PDI was colocalized with NR1 clusters (Fig. 4G). In the distal dendrites, PDI immunoreactive structures were observed as continuous tubular (ER-like) or punctuate (vesicle-like) shapes. Some PDI positive structures contained NR1 immunoreactivity and attached to NR1 positive puncta (Fig. 4G). Together with the inhibitory effects of PDI siRNA and PDI neutralization on seizure susceptibility, these findings suggest that PDI may be involved in the thiol modification of NMDAR in ER as well as on cell surface.

Since NR1 subunit has cysteine residues resembling potential motifs for glutathionylation^11^, we also assessed whether PDI knockdown affects glutathionylation-mediated NMDAR redox signals. However, we could not find the glutathionylation on NR1 subunit in control siRNA- and PDI-siRNA infused animals (Supplementary Fig. 1A). These findings indicate that glutathionylation on NR1 may not influence on thiol modification induced by PDI knockdown. Together with the effect of PDI knockdown on the amount of -SH + -SNO residues in NMDAR subunits, our findings indicate that PDI may participate in NMDAR redox modulation without glutathionylation of thiol modification on NR1.

### PDI knockdown effectively inhibits spontaneous seizure activity in chronic epileptic rats

**Figure 5:**
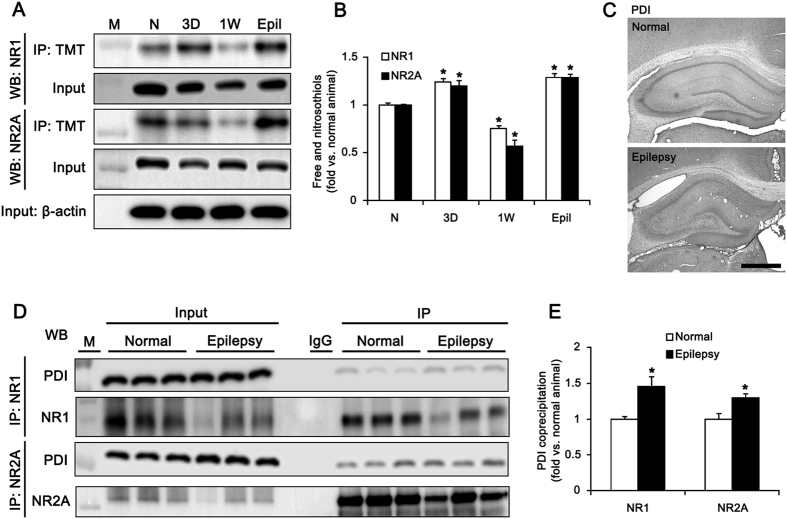
Changed redox status of NMDAR by PDI in epileptic rats. (**A**) Western blot data for the amounts of -SH + -SNO of NR1 and NR2A subunits in non-SE (N), 3 day-post SE (3D), 1 week-post SE (1W) and chronic epileptic (6 week-post SE, Epil) animals. M, molecular weight marker. (**B**) Quantification of the amount of -SH + -SNO on NR1 and NR2A (mean ± S.E.M.; *p < 0.05 vs. normal; n = 7, respectively). The amounts of -SH + -SNO of NR1 and NR2A are increased 3 days after SE, and subsequently recovered to normal normal level 1 week after SE. (**C**) Representative photos for PDI expression in the normal and epileptic hippocampus. Bar = 300 μm. (**D**) Co-immunoprecipitation analyses of NR1 and NR2A interaction with PDI in normal and epileptic animals. M, molecular weight marker. (**E**) The quantitative analyses of co-immunoprecipitation of PDI with NR1 and NR2A in normal and epileptic animals (*p < 0.05 vs. normal; n = 7, respectively). In chronic epileptic animals, the amount of -SH + -SNO of NR1 is increased, as compared to normal animals.

PILO-induced SE shows acute symptomatic period of during 1–3 days (acute phase), which are followed by a silent (latent or epileptogenic) period of 3–4 weeks before the onset of chronic epilepsy^25^,^26^,^27^. Therefore, we investigated the chronological profiles of PDI-mediated reduction of disulfide bonds induced by SE. The amounts of -SH + -SNO of NR1 and NR2A subunits were increased to 1.24- and 1.2-fold of normal level 3 days after SE, respectively (p < 0.05 vs. normal animals; Fig. 5A,B). However, the amounts of -SH + -SNO of NR1 and NR2A subunits were decreased to 0.75- and 0.57-fold of normal level 1 week after SE, respectively (p < 0.05 vs. normal; Fig. 5A,B). In chronic epileptic animals, the amount of -SH + -SNO of NR1 was rebounded to 1.29- and 1.28-fold of normal level, respectively (p < 0.05 vs. normal animals; Fig. 5A,B). Consistent with our previous study^23^, PDI expression was markedly reduced in the CA1 region of chronic epileptic animals due to massive neuronal death. In dentate granule cells, PDI expression in epileptic rats was similar to that in normal animals (Fig. 5C). However, co-precipitation of NMDAR with PDI was increased in epileptic animals by approximately 2.4-fold, as compared to normal animals (p < 0.05; Fig. 5D,E). These findings suggest that PDI-mediated reduction of disulfide bonds may be related to recurrent seizure activity in chronic epileptic animals.

**Figure 6:**
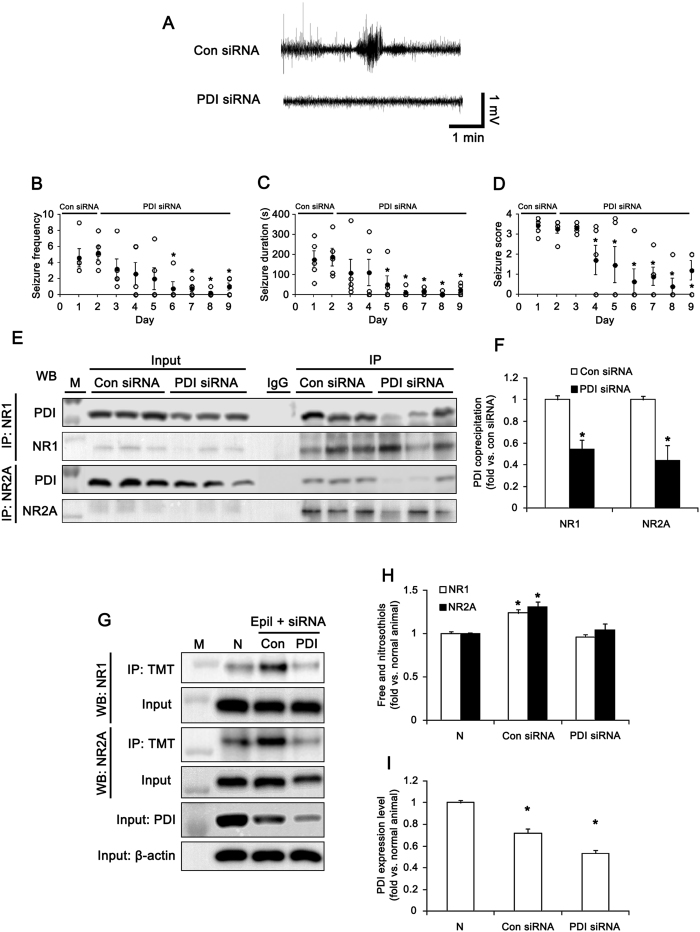
Effect of PDI knockdown on spontaneous seizure activity in epileptic (6 week-post SE) rats. (**A**) Representative EEGs for control siRNA- and PDI siRNA-infused epileptic animals. (**B–D**) Anticonvulsive effect of PDI siRNA on spontaneous seizure activity: (**B**) the mean seizure frequency, (**C**) seizure duration and (**D**) behavioral seizure score (Open circles indicate each individual value. Closed circles indicate mean value. *p < 0.05 vs. control siRNA; n = 5, respectively). PDI knockdown inhibits the spontaneous seizure activity in chronic epileptic rats. (**E**) Co-immunoprecipitation analyses of NR1 or NR2A interaction with PDI in control siRNA- and PDI siRNA-infused epileptic animals. M, molecular weight marker. (**F**) The quantitative analyses of co-immunoprecipitation of PDI bound to NR1 and NR2A (*p < 0.05 vs. control siRNA; n = 7, respectively). (**G**) Western blot data for the amounts of -SH + -SNO on NR1 and NR2A subunits in normal (N), control siRNA- and PDI siRNA-infused epileptic animals. M, molecular weight marker. (**H,I**) Quantification of effects of PDI siRNA on the amount of -SH + -SNO on NR1 and NR2A subunit and PDI expression in epileptic rats (mean ± S.E.M.; *p < 0.05 vs. normal; n = 7, respectively). PDI siRNA infusion reduces the binding of PDI to NMDAR, the amount of -SH + -SNO of NMDAR subunits and PDI expression level in epileptic animals.

To explore the direct role of PDI in the generation of spontaneous seizure activity, we accessed PDI siRNA in epileptic animals. In control siRNA-infused epileptic animals, the mean seizure frequency was 4.6 ± 1.12/recording session (2 h) and the total seizure duration was 175 ± 42.3 s. In addition, behavioral seizure severity (Racine score) was 3.4 ± 0.19. (Fig. 6A–D). PDI siRNA infusion reduced the mean seizure frequency and the total seizure duration to 0.9 ± 0.45/recording session and 15 ± 7.1 s, respectively. PDI siRNA infusion also decreased behavioral seizure score to 1.7 (p < 0.05 vs. control siRNA; Fig. 6A–D). PDI siRNA infusion effectively decreased the binding of PDI to NMDAR, the amount of -SH + -SNO of NMDAR subunits and PDI expression level in epileptic animals (p < 0.05 vs. control siRNA; Fig. 6E–I). Therefore, our findings reveal that PDI-mediated NMDAR redox may play an important role in ictogenesis in chronic epileptic rats.

### PDI knockdown does not induce ER stress

**Figure 7:**
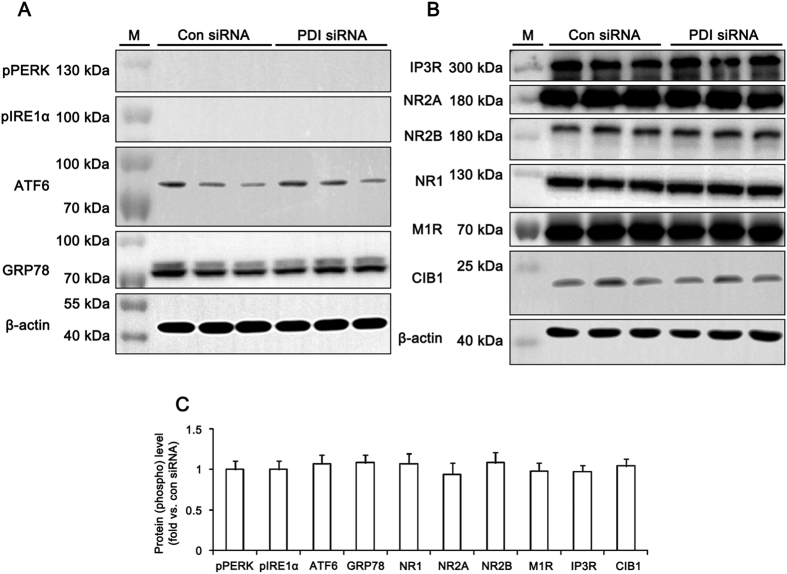
Effects of PDI knockdown on ER stress induction and excitatory receptor expressions. (**A**) Western blot data for ER stress makers including pPERK, pIRE1α, IRE1α and ATF6 at 7 days after PDI siRNA infusion. M, molecular weight marker. (**B**) Western blot data for excitatory receptors including IP3R, NR2A, NR2B, NR1, M1R and CIB1 at 7 days after PDI siRNA infusion. M, molecular weight marker. (**C**) Quantification of effects of PDI siRNA on levels of ER stress markers and receptor expressions (n = 7, respectively). PDI knockdown does not induce ER stress and alterations in excitatory receptor expressions.

ER is a cell organelle to regulate in the glycosylation, folding and assembly of newly synthesized proteins. Stressful conditions such as oxidative stress, hypoxia and changed glucose metabolism disturb ER function due to the accumulation of unfolded proteins or changes in Ca^2+^ homeostasis^28^. ER stress induces the expressions of chaperones, attenuation of protein translation and activation of ER-associated degradation through the activation of ER sensor proteins including protein kinase RNA (PKR)-like ER kinase (PERK), inositol-requiring protein 1-α (IRE1α), activating transcription factor 6 (ATF6) and glucose-regulated protein 78 (GRP78)^29^,^30^. Since PDI is one of the initial endoplasmic reticulum (ER) chaperones, which is involved in the regulation of ER stress^17^,^18^,^31^, we investigated whether PDI siRNA leads to ER stress. In the present study, the levels of phospho (p)-PERK (pPERK), pIRE1α, ATF6 and GRP78 were unaltered by PDI siRNA infusion over a 7-day period (Fig. 7A–C), although tunicamycin (an ER stress inducer, 120 μM) increased pPERK, pIRE1α and GRP78 expression in the hippocampus (Supplementary Fig. 1B,C). These findings indicate that PDI knockdown may not induce ER stress under physiological condition.

### PDI siRNA does not affect the expression levels of NMDAR, M1R, IP3R and CIB1

Because the M1R and other glutamate receptor mediate PILO-induced seizure activity^32^, we investigated whether the influences of PDI knockdown on various excitatory receptor expressions affect the PILO-induced seizure threshold. There was no difference in M1R and NMDAR expression levels in the hippocampus between control- and PDI siRNA-infused animals (Fig. 7B,C). Since PILO induces seizure activity via IP3R activation^32^,^33^,^34^, we also investigated expression levels of IP3R. PDI knockdown did not affect IP3R expression level as compared to control siRNA (Fig. 7B,C). On the other hand, PDI limits the duration of IRE1α activity^35^, which regulates calcium and integrin-binding protein 1 (CIB1, a Ca^2+^-binding IP3 receptor inhibitor)-mediated inhibition of IP3R receptor from ER^36^. Thus, we tested whether PDI siRNA affects the expression levels of CIB1, which affects the seizure susceptibility. In the present study, PDI siRNA did not affect CIB1 expression level (Fig. 7B,C). These findings indicate that PDI knockdown may increase the seizure threshold in response to PILO independent of altered receptor expression level.

### PDI knockdown reduces PILO-induced seizure susceptibility independent of GABAergic or glutamatergic transmission

**Figure 8:**
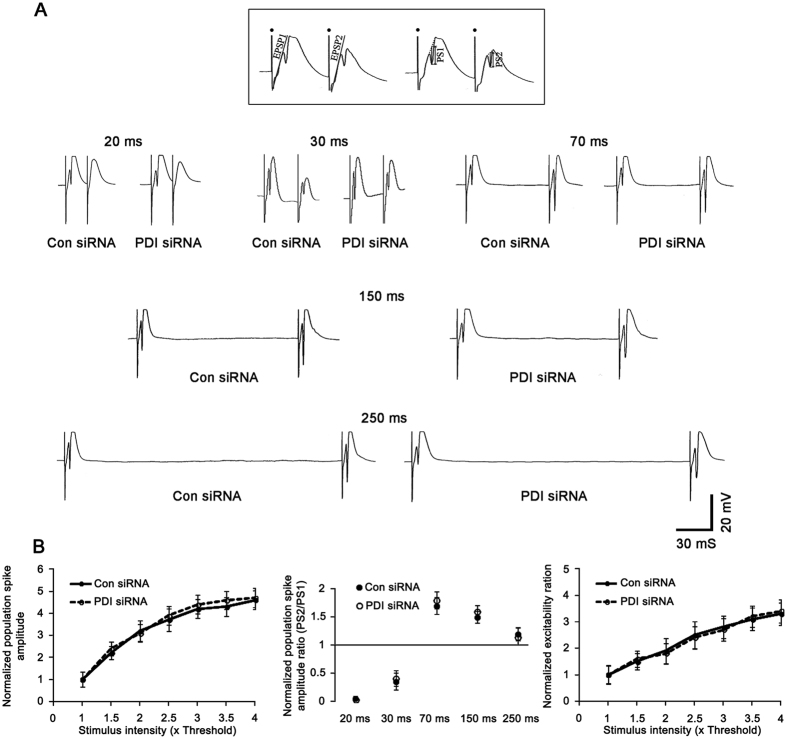
Effect of PDI knockdown on basal neurotransmission in the dentate gyrus at 7 days after PDI siRNA infusion. (**A**) Representative paired-pulse responses in control siRNA- and PDI siRNA-infused animals. The inset at top shows the measurement of fEPSP slope (EPSP) and population spike amplitude (PS). Filled circles indicate stimulus artifacts. (**B**) Quantification of effects of PDI siRNA on IO curve, normalized population spike amplitude ratios and normalized excitability ratio in the dentate gyrus (n = 7, respectively). PDI knockdown does not affect GABAergic or glutamatergic transmission in basal condition.

To determine whether PDI knockdown reduces PILO-induced seizure susceptibility via alteration in GABAergic or glutamatergic transmission, we investigated the effect of PDI siRNA infusion on the paired-pulse responses of the dentate gyrus *in vivo*. There was no difference in the IO curve between control siRNA- and PDI siRNA-infused animals (Fig. 8A,B). Both control siRNA- and PDI siRNA-infused animals showed a strong paired-pulse inhibition at 20 and 30 ms interstimulus intervals (fast inhibition), a noteworthy paired-pulse facilitation at 70 and 150 ms intervals, and a less pronounced paired-pulse inhibition at 250 ms interval (late inhibition). There were no differences in the normalized population spike amplitude ratio at any interstimulus interval between control siRNA- and PDI siRNA-infused animals (Fig. 8A,B). Since a population spike reflects a synchronous postsynaptic discharge and fEPSP slope is relevant to presynaptic excitability^37^, we also analyzed the excitability ratio (the ratio of the population spike amplitude to fEPSP slope), so-called fEPSP slope-population spike amplitude (E-S) coupling, as an index of synaptic efficacy and excitability of the dentate gyrus^38^. There was no difference in the normalized excitability ratio between control siRNA- and PDI siRNA-infused animals (Fig. 8A,B). These findings indicate that PDI knockdown may reduce PILO-induced seizure susceptibility without change in GABAergic or glutamatergic transmission.

## Discussion

The major findings in the present study are that PDI is one of endogenous reducing agents for disulfide bonds of cysteine residues on NMDAR subunits, and plays an important role in the modulation of ictogenesis and seizure susceptibility.

NMDAR controls diverse functions such as neuronal development, synaptic plasticity and memory. However, NMDAR hyperactivation leads to neuronal cell death in a variety of acute and chronic neurological diseases including epilepsy^39^. Therefore, tight regulation of NMDAR activity is crucial for maintenance of neuronal function and survival. Together with phosphorylation^40^,^41^, redox modulation of disulfide bonds on cysteine residues is one of the chemical modifications to regulate NMDAR activity^10^,^11^,^15^. In NMDAR, six of the cysteine residues (Cys79 and Cys308; Cys744 and Cys798 of NR1; Cys87 and Cys320 of NR2A) are involved in redox modulation and disulfide bond formations (HS + SH → S-S) by endogenous oxidizing agents (GSSG or lipoic acid)^10^,^11^,^15^. In the present study, PDI increased thiols on full-length recombinant NR1 protein *in vitro*. Furthermore, PDI siRNA inhibited PDI activity accompanied by the reductions in the amounts of -SH + -SNO residues in NR1 and NR2A subunits *in vivo*. These finding indicate that PDI may bind NR1 and NR2A subunits to modulate the disulfide bond formation on NMDAR under physiological condition.

The oxidation on cysteine residues of NMDAR decreases the number of channel openings, while GSH and dihydrolipoic acid enhance NMDAR activity by reducing disulfide bonds (sulfhydration; S-S → HS + SH)^42^,^43^. Under physiological and pathophysiological conditions, thus, endogenous oxidizing agents limit excessive NMDAR activation^14^,^15^. Indeed, Di Maio *et al*.^44^ report that PILO-induced seizure results in the loss of NMDAR-induced Ca^2+^ influx accompanied by increase in total protein thiol oxidation (increase in disulfide bonds), and speculate that disulfide bonds on NMDAR could be a potentially compensatory response for preventing Ca^2+^-mediated neurotoxicity via unknown mechanism. Recently, we have reported that PDI expression is transiently increased in the hippocampus following SE^23^. Since PDI is an ER chaperone^17^,^18^ and salubrinal (an ER stress inhibitor) attenuates kainic acid-induced seizure activity^45^,^46^, we postulated that up-regulation of PDI expression might be one of the adaptive responses for ER stress^17^,^18^,^23^,^31^ to increase seizure threshold in response to PILO. Unexpectedly, the present study demonstrates that PDI siRNA, DTNB and bacitracin decreased seizure susceptibility in response to PILO, accompanied by the increases in disulfide bonds of cysteine residues on the NMDAR subunits under physiological condition. Furthermore, PDI knockdown reduced in spontaneous seizure activity, the binding of PDI with NMDAR subunits and the amount of -SH + -SNO of cysteine residues on the NR1 and NR2A subunits in epileptic rats. Based on the characteristics of catalyzing disulfide bond formation^19^,^20^,^21^,^22^, these data provide mechanistic insight into sulfhydration (reduction) of disulfide bonds on NMDAR subunits by PDI, which may increase seizure susceptibility and involve ictogenesis.

NMDAR is synthesized in the rough ER of the soma, packaged in the Golgi apparatus, and are subsequently transported to the spine apparatus (a specialized form of ER) at the base of dendritic spines or the extrasynaptic membrane. Thereafter, NMDAR is fused with the synaptic or extrasynaptic membrane^5^. In the present study, PDI immunoreactivity was colocalized with NR1 clusters in the primary dendrite of hippocampal neurons. In the distal dendrites, PDI positive structures showed continuous tubular ER-like shapes or punctuate vesicle-like shapes, which contained or attached to NR1 positive puncta. Furthermore, the present study showed that PDI neutralization effectively reduced seizure susceptibility in response to PILO. Since PDI is localized in various cell organelles and on the cell surface^19^,^20^,^21^,^22^, our findings suggest that PDI may participate in the thiol modification of NR1 and NR2A subunits in ER as well as cell surface.

PILO is one of the agonists for M1R, which induces accumulation of intracellular IP3 concentrations, and in turn facilitates the intracellular Ca^2+^ release via IP3R^32^,^33^. Thus, IP3R antagonism inhibits PILO-induced seizure activity^34^. Since PDI is an ER protein^17^,^18^, it is plausible that PDI knockdown would inhibit M1R-mediated IP3R activation. However, the co-precipitations of PDI with M1R and IP3R were rarely observed in the present study. Since increased IP3 production elicited by M1R can lead to NMDAR activation without a concurrent intracellular Ca^2+^ release^44^,^47^, our findings suggest that effect of PDI knockdown on the thiol modification on NMDAR may be independent of M1R-mediated IP3R activation.

Although NMDAR inhibition is a potential therapeutic antiepileptic strategy, therapeutic doses of NMDAR antagonists show the various adverse effects^48^,^49^,^50^. In contrast to NMDAR antagonist, oxidation of the NMDAR redox site inhibits seizure epileptiform activity without blocking physiological NMDAR functions *in vitro*^51^. Similarly, the present study shows that PDI knockdown did not affect basal GABAergic or glutamatergic transmission *in vivo*. Indeed, PDI siRNA could not alter the expression levels of NMDAR, IP3R and M1R. Furthermore, PDI knockdown did not affect the phosphorylations and expressions in any ER stress markers. These findings indicate that PDI knockdown may not induce alteration in basal neurotransmission and ER stress under physiological condition.

In conclusion, the present data show that PDI knockdown inhibited the reduction of disulfide bonds on NMDAR subunits, resulting in decreased seizure susceptibility in response to PILO. Moreover, spontaneous seizure activity in chronic epileptic animals was ameliorated by PDI siRNA infusion. Therefore, our findings suggest that PDI may be one of the attractive therapeutic targets for epilepsy, which avoids a possible adverse effect on physiological receptor functionality/expression without ER stress induction.

## Methods

### Experimental animals and chemicals

Male Sprague-Dawley (SD) rats (7 weeks old) were used in the present study. The colony room was maintained at 22 ± 2 °C, 55 ± 5% and a 12:12 light/dark cycle with lights, and food and water *ad libitum* throughout the experiments. All experimental protocols described below were approved by the Institutional Animal Care and Use Committee of Hallym University (Chuncheon, South Korea) and all efforts were made to minimize animal suffering. The procedures involving animals and their care were conducted in accord with our institutional guidelines that comply with NIH Guide for the Care and Use of Laboratory Animals (NIH Publications No. 80–23, 1996). All reagents were obtained from Sigma-Aldrich (St. Louis, MO, USA), except as noted.

### Surgery

Implantation Surgery for a brain infusion kit and an electrode was performed according to our previous studies^52^,^53^. Briefly, animals were anesthetized with isoflurane (3% induction, 1.5–2% for surgery and 1.5% maintenance in a 65:35 mixture of N_2_O:O_2_). Throughout surgery, the animals were positioned over a heated pad, and core temperature was monitored and maintained between 37 and 38 °C. A brain infusion kit 1 (Alzet, Cupertino, CA, USA) was implanted into the right lateral ventricle (1 mm posterior; 1.5 mm lateral; 3.5 mm depth from bregma) and connected to an osmotic pump (1007D, Alzet, Cupertino, CA, USA) containing (1) control siRNA, (2) PDI siRNA, (3) vehicle, (4) DTNB (1 mM), (5) bacitracin (2 mM), (6) control IgG (50 ug/ml, Abcam, Cambridge, UK), (7) anti-PDI IgG (clone RL90, 50 ug/ml, Abcam, Cambridge, UK, Cat: ab2792) and (8) tunicamycin (an ER stress inducer, 120 μM)^52^,^53^. A set of four on-target *PDI* rat siRNAs were applied in the pilot study. Among them, a 21-nt siRNA sequence targeting *PDI* corresponding to coding region (5′→3′): sense: CUGCAAAACUGAAGGCAGAUU, and antisense: UCUGCCUUCAGUUUUGCAGUU was selected as the best probe and used for the final experiments. A non-silencing RNA was used as the control siRNA. The pump was subcutaneously placed subcutaneously in the interscapular region. Some animals were also implanted by monopolar stainless steel electrode (Plastics One, Roanoke, VA, USA) implanted into the left dorsal hippocampus (3.8 mm posterior; 2.0 mm lateral; 2.6 mm depth from bregma).

### Paired-pulse responses

Seven days after surgery, animals were anesthetized (urethane, 1.5 g/kg, i.p.), removed the infusion kit and osmotic pump, and placed in a stereotaxic frame. Rectal temperature was maintained at 36.5 ± 0.5 °C using a homeothermic temperature control unit. The skull was exposed and two small holes were drilled over the dentate gyrus (3.8 mm posterior; 2.5 mm lateral, 2.9 mm depth from bregma) and the angular bundle (4.2 mm lateral; 3.0 mm depth from bregma). A monopolar recording electrode was positioned in the dentate gyrus, and a bipolar stimulating electrode was positioned in the angular bundle. Electrode depths were set by optimizing the evoked responses. The reference electrode was placed in the posterior cranium over the cerebellum. Signal was amplified (1000 × ), filtered (1 Hz to 3 kHz) (DAM 80 differential amplifier, WPI, Sarasota, FL, USA), digitized, recorded and analyzed using LabChart Pro v7 software (AD Instruments, NSW, Australia). After ensuring a steady-state baseline response, an input–output (I/O) function was obtained by systematic variation of the stimulus current (100–1000 μA) in order to evaluate synaptic potency. To analyze changes in evoked response, all of the population spike amplitudes and the field excitatory postsynaptic potential (fEPSP) slope measurements during recording session were normalized by the averages of the population spike amplitude and fEPSP slope of the first response during the baseline measurement. To measure the efficiency of glutamatergic synaptic transmission in the dentate gyrus, the excitability ratio was calculated as the population spike amplitude vs. the fEPSP slope in the first response^38^,^54^.

### SE induction

SE was induced by intraperitoneal injection of pilocarpine as described previously^52^,^53^. Briefly, rats were pretreated with an intraperitoneal injection of LiCl (127 mg/kg i.p) 24 h before the PILO treatment. Animals were intraperitoneally (i.p) treated with pilocarpine (30 mg/kg) 20 min after atropine methylbromide (5 mg/kg i.p.). PILO injection resulted in stereotypical behavioral responses, which included the following: akinesia, staring, salivation, facial automatisms, slight tremors and head bobbing. These behavior responses built up progressively into motor limbic seizures that recurred repeatedly and rapidly developed into SE characterized by forelimb clonus and tonic-clonic seizures with loss of righting reflexes. SE was defined by continuous or intermittent seizures without full recovery between seizures. Control animals received an equal volume of normal saline instead of PILO after the pretreatment with atropine methylbromide. Diazepam (Valium; Hoffman la Roche, Neuilly sur-Seine, France; 10 mg/kg, i.p.) was administered 2 h after onset of SE and repeated, as needed. Animals were video-monitored 8 h a day for general behaviour and occurrence of spontaneous seizures by 6 weeks after SE. Rats showing spontaneous recurrent seizures were used as chronic epileptic animals.

### Analysis of seizure susceptibility in response to PILO

Seven days after an osmotic pump implantation, SE was induced by PILO injection as described previously^54^. After baseline recording for at least 30 min, rats were injected with saline or PILO. EEG signals were digitized (1000 Hz) and analyzed using LabChart Pro v7 software (AD Instruments, NSW, Australia). Time of seizure onset was defined as the time point showing paroxysmal depolarizing shift, defined as lasting more than 3 s and consisting of a rhythmic discharge between 4 and 10 Hz with amplitude of at least two times higher than the baseline EEG^34^. Total power was measured during the 2-h recording session from each animal by LabChart Pro v7 (AD Instruments, NSW, Australia). Spectrograms were automatically calculated using a Hanning sliding window with 50% overlap.

### EEG analysis and quantification of behavioral seizure activity in chronic epileptic rats

We applied a modified protocol for the effect of PDI knockdown on spontaneous seizure activity in chronic epileptic rats based on Ko and Kang and Glien *et al*.^53^,^55^. After baseline seizure activity (control siRNA treatment) was determined over 2 days, *PDI* siRNA was administered over a 7-day period using an osmotic pump (1007D, Alzet, Cupertino, CA, USA). Between trials, the minipump was changed out for another minipump filled with another mixture under isoflurane anesthesia. Every day during the experiment, spontaneous seizure activity was recorded by video-EEG monitoring with 2 h of recording per day at the same time. EEG analysis was performed by LabChart Pro v7 (AD Instruments, NSW, Australia). Behavioral seizure severity was also evaluated according to Racine’s scale^56^: 1, immobility, eye closure, twitching of vibrissae, sniffing, facial clonus; 2, head nodding associated with more severe facial clonus; 3, clonus of one forelimb; 4, rearing, often accompanied by bilateral forelimb clonus; and 5, rearing with loss of balance and falling accompanied by generalized clonic seizures.

### Co-immunoprecipitation

The hippocampal tissues were lysed in radioimmune precipitation buffer (RIPA: 50 mM Tris–HCl pH 8.0; 1% Nonident P-40; 0.5% deoxycholate; 0.1% SDS) containing protease inhibitor cocktail (complete, Roche Diagnostics GmbH, Penzberg, Germany), phosphatase inhibitor cocktail (PhosSTOP^®^, Roche Diagnostics GmbH, Penzberg, Germany) and 1 mM sodium orthovanadate. Protein concentrations were determined by BCA protein assay (Pierce, Rockford, IL, USA) and equal amounts of total proteins were precipitated with the appropriate primary antibodies and protein G sepharose at 4 °C overnight. Beads were collected by centrifugation, eluted in 2 × SDS sample buffer and boiled at 95 °C for 5 min. Next, Western blotting was performed according to standard procedures (see below).

### Detection of PDI in CSF

CSF samples were obtained from cisterna magna using the stereotaxic instruments and collected in 0.2 ml microtubes. CSF samples were concentrated with vacuum centrifugation, and dissolved in RIPA buffer for western blot analysis and PDI assay.

### Measurement of PDI activity

The hippocampal tissues were lysed in RIPA buffer, and protein concentrations were determined by BCA protein assay (Pierce, Rockford, IL, USA). Equal amounts of total proteins (1 mg) were used to measure PDI activity with PROTEOSTAT^**®**^ PDI assay kit (Enzo life sciences, Farmingdale, NY, USA) according to the manufacturer’s protocol.

### *In vitro* thiol reductase assay

Modified *in vitro* thiol reductase assay using full-length recombinant human NR1 protein (Novus biologicals, Littleton, CO, USA, Cat: H00002902-G01) was performed as described previously^57^. Briefly, 0.5 μg recombinant human NR1 protein (in 25 mM Tris-HCl of pH 8.0 containing 2% glycerol, Novus biologicals, Littleton, CO, USA) was incubated with recombinant human PDI (0.5 μg in 50 mM Tris, pH 7.5 containing 150 mM NaCl, 1 mM DTT, 1 mM EDTA and 5% glycerol, Enzo life sciences, Farmingdale, NY, USA, Cat: ADI-SPP-891) at room temperature for 2 h. Control test was carried out with equal amount of 50 mM Tris buffer (pH 7.5 containing 150 mM NaCl, 1 mM DTT, 1 mM EDTA and 5% glycerol) instead of PDI. Thereafter, the amounts of free thiols on NR1 protein and the presence of PDI were confirmed by modified biotin switch assay and western blot using TMT, NR1 and PDI antibody on the same membrane (see below).

### Measurement of free- and nitrosothiols

Modified biotin switch assay was performed with the S-nitrosylation Western Blot Kit (Thermo Fisher Scientific, Waltham, MA, USA) according to the manufacturer’s protocol (Fig. 3C). Briefly, lysates were reacted with ascorbate in HENS buffer for specific labeling with iodoTMTzero reagents. Protein labeling can be confirmed by Western blot using TMT antibody. Thereafter, TMT-labeled proteins were purified by Anti-TMT Resin, eluted by TMT elusion buffer, and identified by Western blot according to standard procedures (see below).

### Western blot

Under urethane anesthesia (1.5 g/kg, i.p.), the hippocampus was dissected out and homogenized in lysis buffer (50 mM Tris containing 50 mM 4-(2-hydroxyethyl)-1-piperazineethanesulfonic acid (pH 7.4), ethylene glycol tetraacetic acid (pH 8.0), 0.2% Tergitol type NP-40, 10 mM ethylenediaminetetraacetic acid (pH 8.0), 15 mM sodium pyrophosphate, 100 mM β-glycerophosphate, 50 mM NaF, 150 mM NaCl, 2 mM sodium orthovanadate, 1 mM phenylmethylsulfonyl fluoride, and 1 mM dithiothreitol). Total protein content was measured by BCA protein assay kit. Western blotting was performed according to standard procedures. The primary antibodies were mouse anti-PDI (1:1,000, clone RL90, Abcam, Cambridge, UK, Cat: ab2792), rabbit anti-NR1 (1:1,000, clone 1.17.2.6, Millipore, Bedford, MA, USA, Cat: AB9864), rabbit anti-NR2A (1:1,000, Thermo Fisher Scientific, Waltham, MA, USA, Cat: OPA1-04021), rabbit anti-NR2B (1:1,000, Millipore, Bedford, MA, USA, Cat: AB1557P), rabbit anti-IP3R (1:1,000, Abcam, Cambridge, UK, Cat: ab5804), rabbit anti-M1R (1:200, Millipore, Bedford, MA, USA, Cat: AB5164), rabbit anti-pPERK (1:500, Biorbyt, San Francisco, CA, USA, Cat: orb6693), rabbit anti-pIRE1α (1:1,000, Thermo Fisher Scientific, Waltham, MA, USA, Cat: PA1-16927), rabbit anti-ATF6 (1:5,000, Proteintech, Seoul, South Korea, Cat: 24169-1-AP), rabbit anti-CIB1 (1:500, Proteintech, Seoul, South Korea, Cat: 11823-1-AP). The rabbit anti-β-actin primary antibody (1:5,000, clone AC-74, Sigma-Aldrich, St. Louis, MO, USA, Cat: A5316) was used as internal reference. The signals were scanned and quantified on ImageQuant LAS4000 system (GE healthcare life sciences, Pittsburgh, PA, USA). The values of each sample were normalized with the corresponding amount of β-actin.

### Immunohistochemistry

Rats were anesthetized with urethane anesthesia (1.5 g/kg, i.p.) and perfused transcardially with 4% paraformaldehyde in 0.1 M phosphate buffer (PB, pH 7.4). Brains were post-fixed in the same fixative overnight and then cryoprotected and sectioned at 30 μm with a cryostat. Free-floating coronal sections were incubated in PDI antibody in PBS containing 0.3% Triton X-100 overnight at room temperature. Tissue sections were developed in 3,3′-diaminobenzidine in 0.1 M Tris buffer and mounted on gelatin-coated slides. Some sections were incubated with mixture of NR1 and PDI antibody in PBS containing 0.3% Triton X-100 overnight at room temperature. Thereafter, sections were visualized with Cy2- and Cy3-conjugated secondary antibody. Immunoreaction was observed using an Axio Scope microscope (Carl Zeiss Inc., Oberkocken, Germany) or a confocal laser scanning microscope (LSM 710, Carl Zeiss Inc., Oberkocken, Germany). To establish the specificity of the immunostaining, a negative control test was carried out with preimmune serum instead of the primary antibody. No immunoreactivity was observed for the negative control in any structures. All experimental procedures in this study were performed under the same conditions and in parallel.

### Statistical analysis

Quantitative data are expressed as mean ± standard error of the mean. Data are analyzed by Student *t*-test or ANOVA followed by Newman–Keuls post-hoc test. A p < 0.05 is considered to be statistically different.

## Additional Information

**How to cite this article**: Kim, J. Y. *et al*. PDI regulates seizure activity via NMDA receptor redox in rats. *Sci. Rep.* **7**, 42491; doi: 10.1038/srep42491 (2017).

**Publisher's note:** Springer Nature remains neutral with regard to jurisdictional claims in published maps and institutional affiliations.

## Supplementary Material

Supplementary Information
