# Supplementary material for: PDI regulates seizure activity via NMDA receptor redox in rats
Source: Sci Rep. 2017 Feb 15;7:42491. doi: 10.1038/srep42491 (PMC5309844; doi:10.1038/srep42491)
Supplement: Supplementary Information [file srep42491-s1.pdf]

Supplementary information

PDI regulates seizure activity via NMDA receptor redox in rats

Ji Yang Kim[*], Ah-Rhem Ko[*], Hye-Won Hyun, Su-Ji Min, Ji-Eun Kim[**]

Department of Anatomy and Neurobiology, Institute of Epilepsy Research, College of Medicine, Hallym University, Chuncheon 24252, South Korea.

**** Address correspondence to:** Dr. Ji-Eun Kim, Department of Anatomy and Neurobiology, Institute of Epilepsy Research, College of Medicine, Hallym University, Chuncheon, Kangwon-Do, 24252, South Korea; jieunkim@hallym.ac.kr

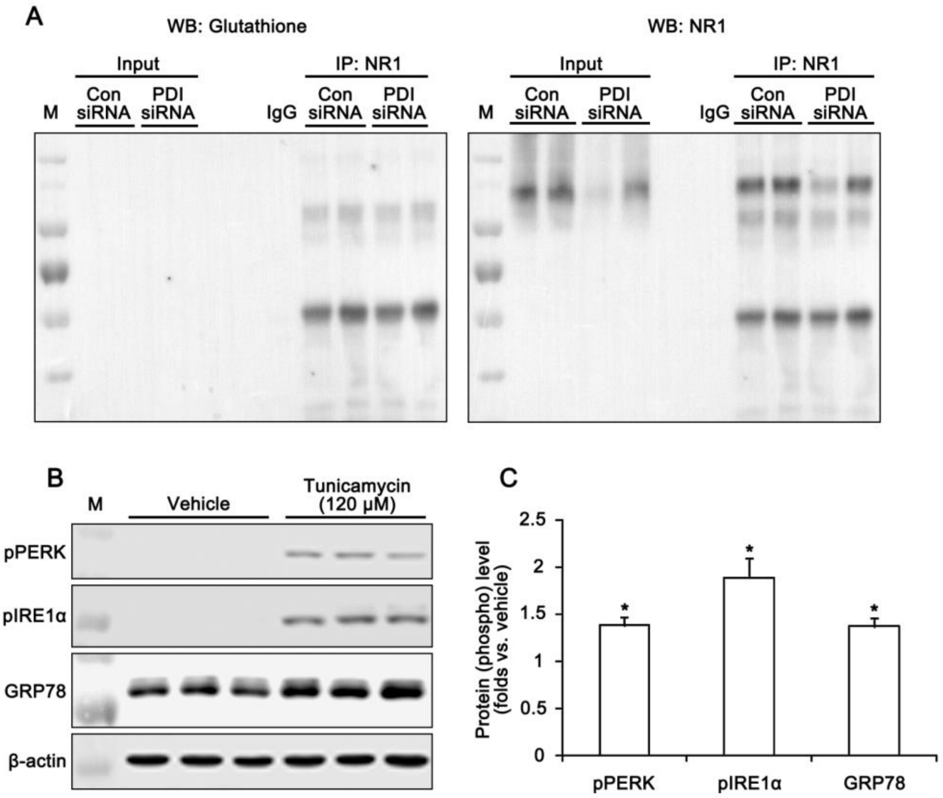

Supplementary Figure 1. (A) Effect of PDI knockdown on glutathionylation-mediated NMDAR redox signals. There is the glutathonylation on NR1 subunit in control siRNA- and PDI siRNA-infused animals. (B) Effect of tunicamycin on ER stress. Tunicamycin increases pPERK, pIRE1α and GRP78 expression in the rat hippocampus, as compared to vehicle (mean ± S.E.M.; $*p < 0.05$ vs. normal; n = 5, respectively).

Fig. 1B

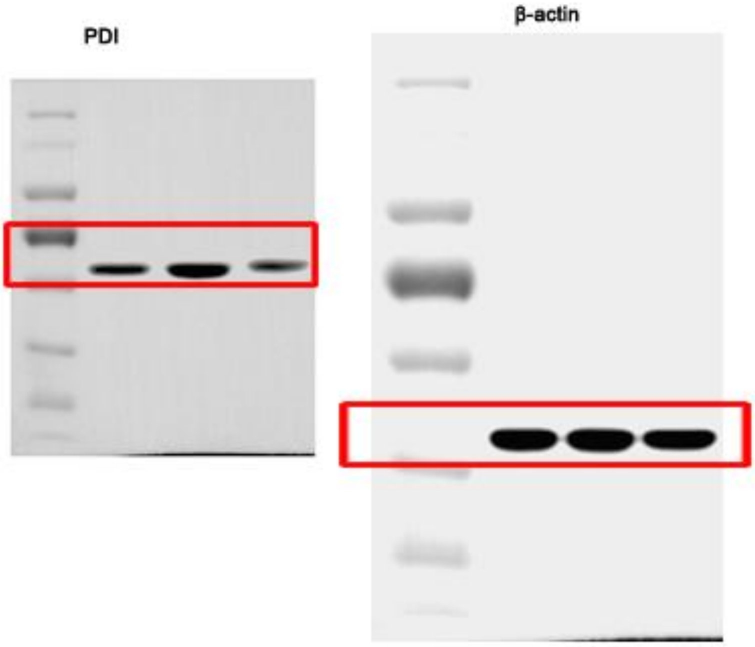

Supplementary Figure 2. Full-length gel images of western blot data in Figure 1. The cropped parts of western blots are indicated with boxes.

Fig. 2A.

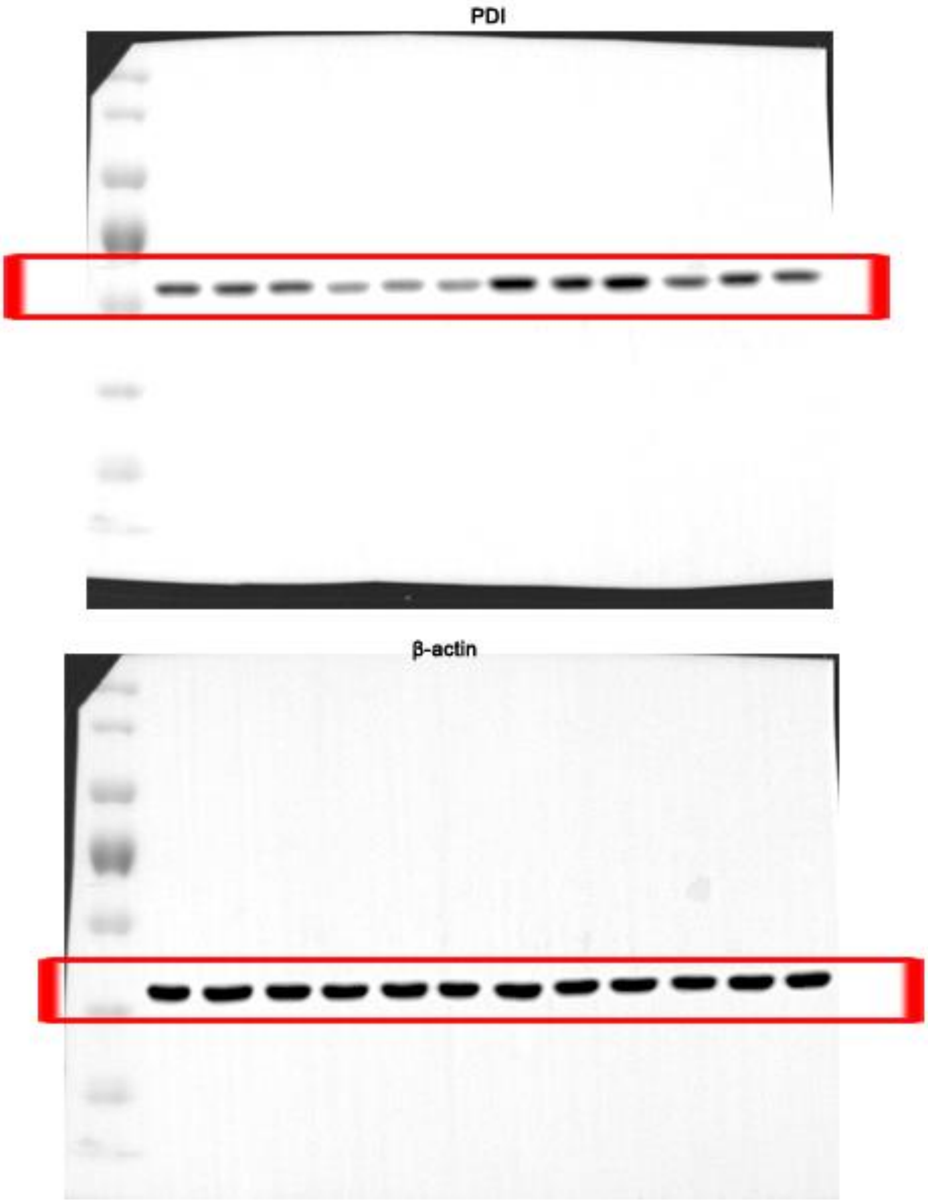

Supplementary Figure 3. Full-length gel images of western blot data in Figure 2. The cropped parts of western blots are indicated with boxes.

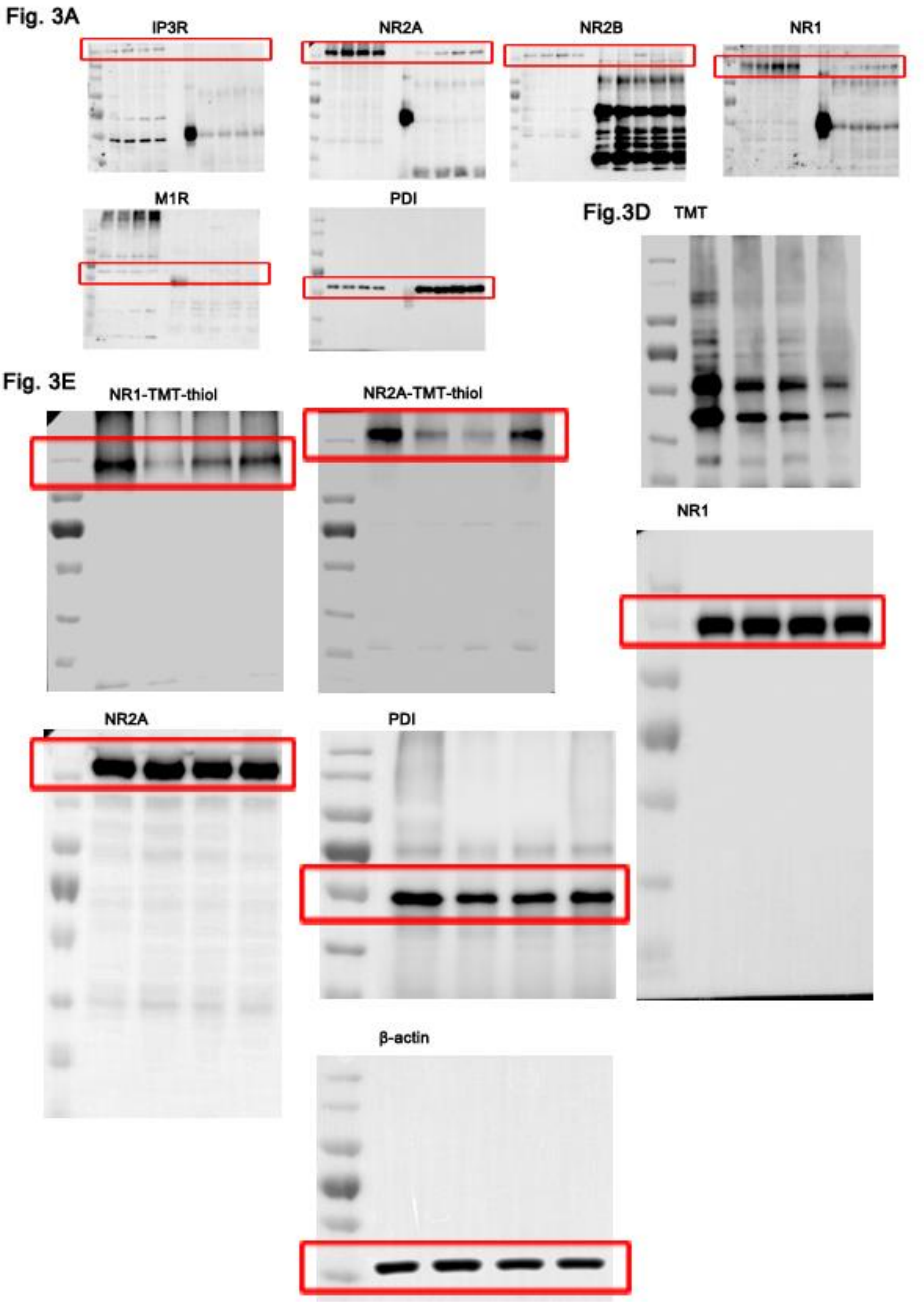

Supplementary Figure 4. Full-length gel images of western blot data in Figure 3. The cropped parts of western blots are indicated with boxes.

Fig. 4D

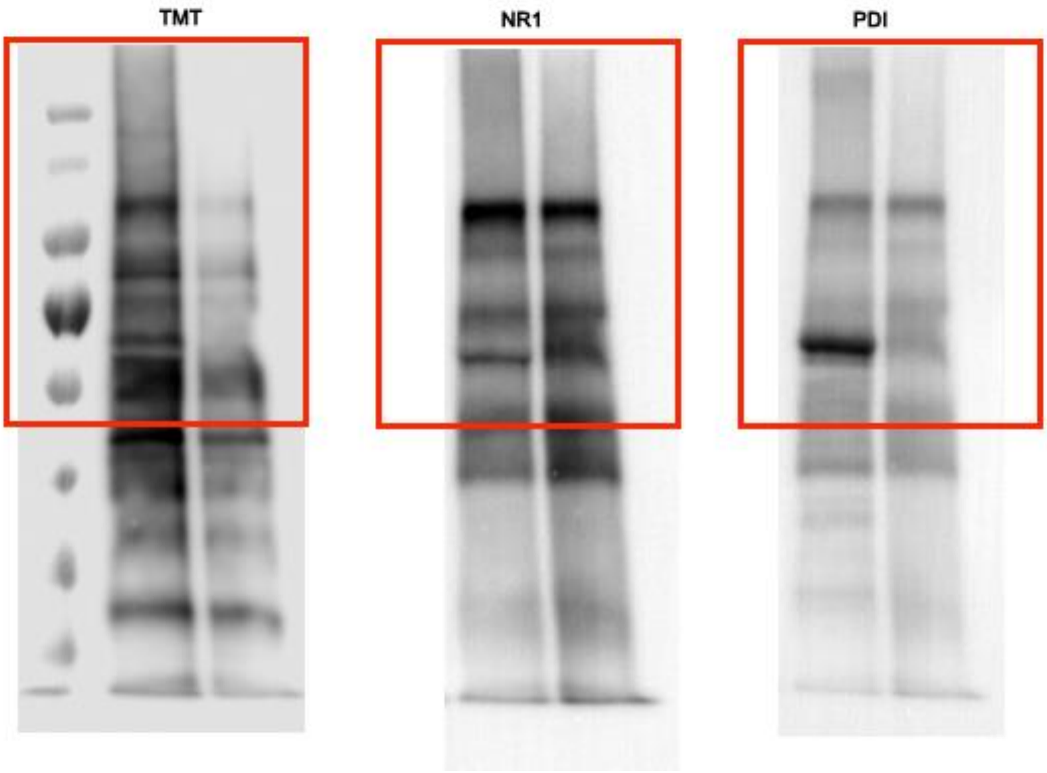

Supplementary Figure 5. Full-length gel images of western blot data in Figure 4. The cropped parts of western blots are indicated with boxes.

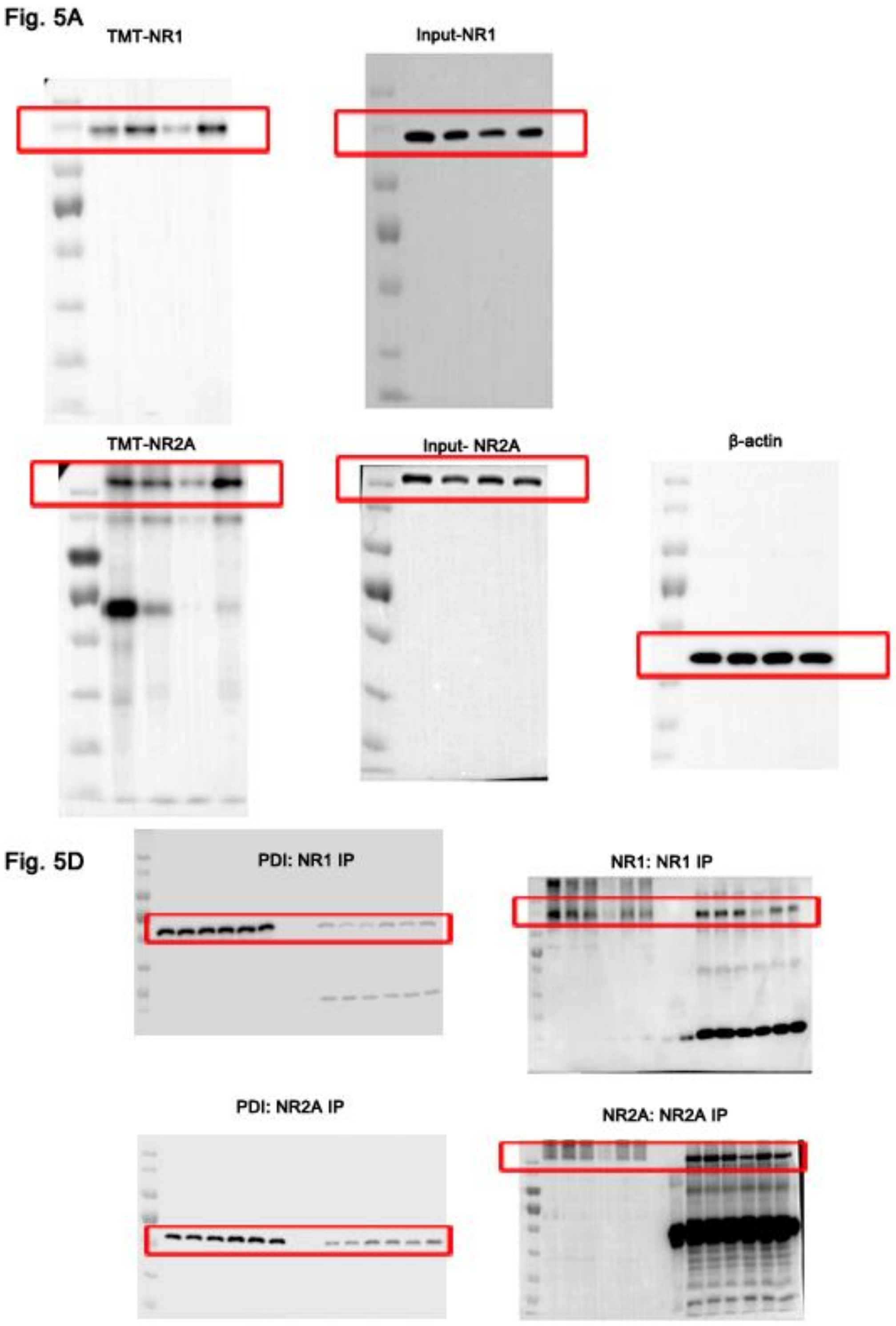

Supplementary Figure 6. Full-length gel images of western blot data in Figure 5. The cropped parts of western blots are indicated with boxes.

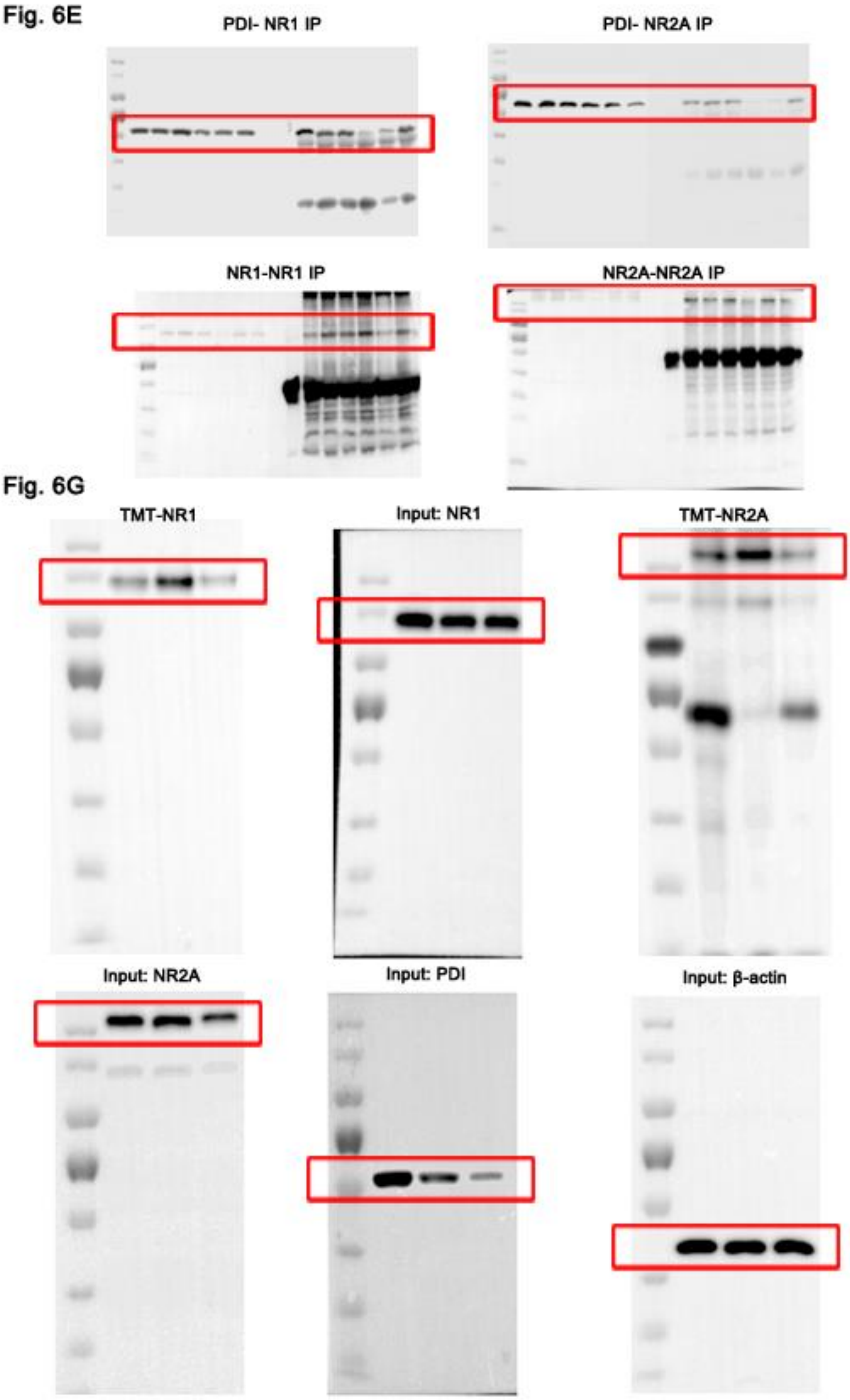

Supplementary Figure 7. Full-length gel images of western blot data in Figure 6. The cropped parts of western blots are indicated with boxes.

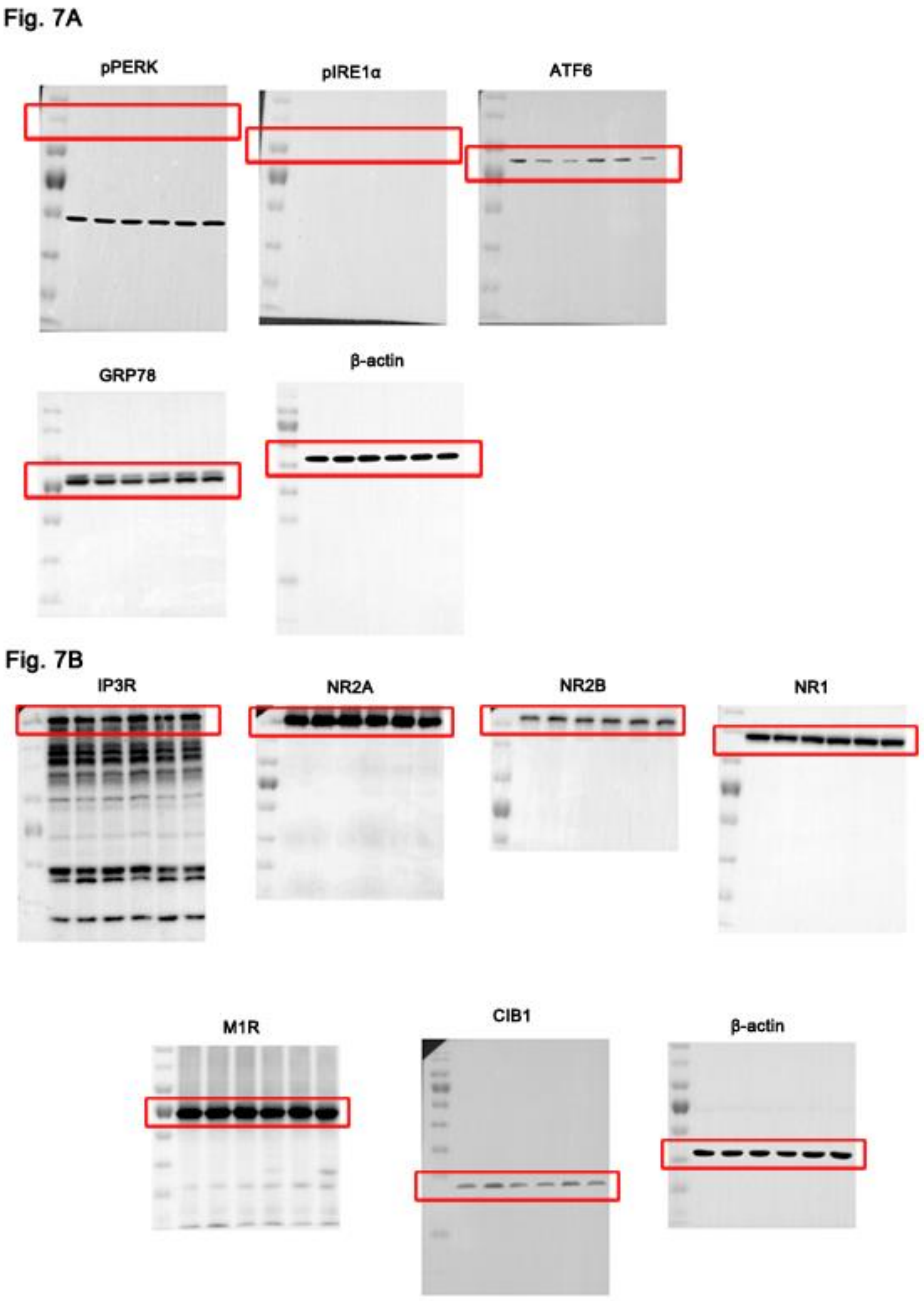

Supplementary Figure 8. Full-length gel images of western blot data in Figure 7. The cropped parts of western blots are indicated with boxes.

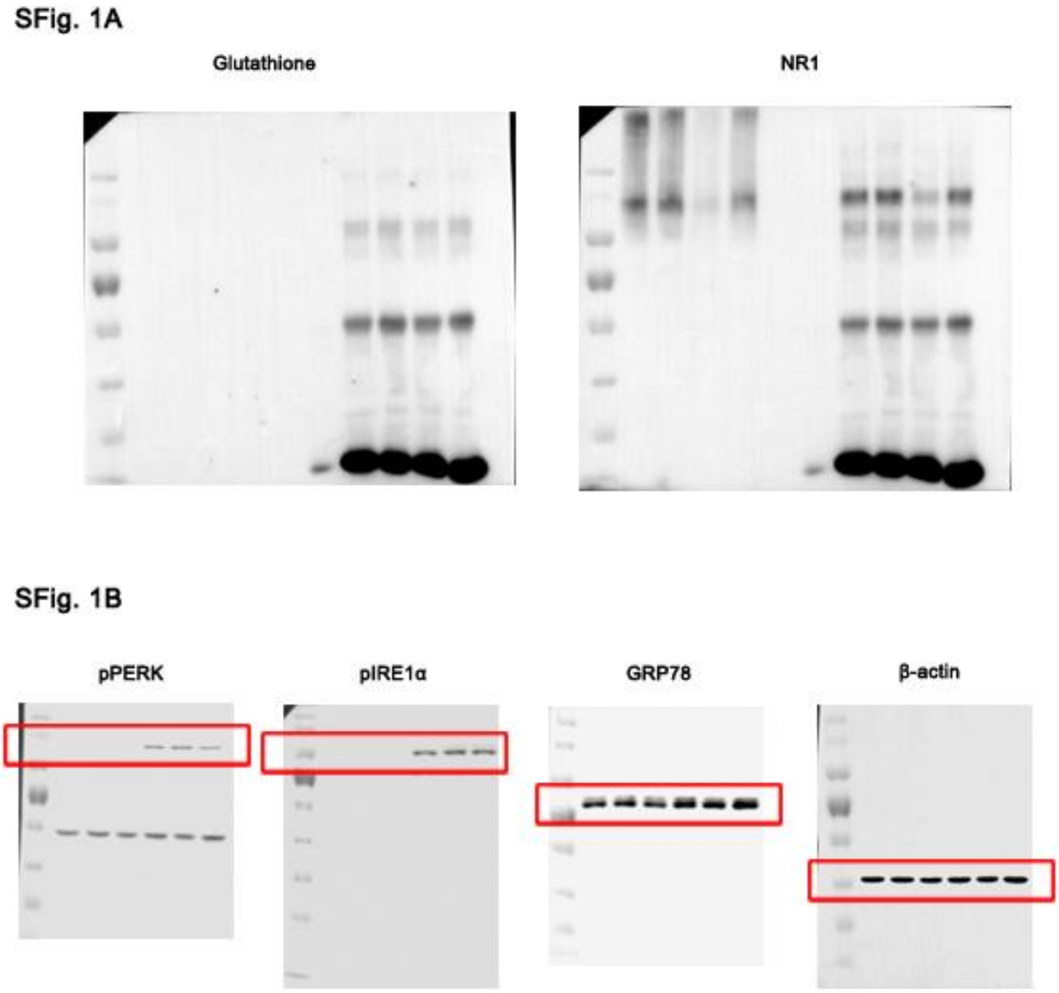

Supplementary Figure 9. Full-length gel images of western blot data in Supplementary figure 1. The cropped parts of western blots are indicated with boxes.
